# Cluster analysis and risk prediction model construction for antisynthase syndrome-associated interstitial lung disease based on clinical, imaging, and antibody characteristics

**DOI:** 10.3389/fmed.2026.1798039

**Published:** 2026-04-01

**Authors:** Jin Zhang, Wenfeng Gao, Baoting Chao, Minghua Huang, Yuyu Yang, Ying Zhang, Rongzhen Xia, Yiting Lin, Kaining Gao, Wei Xu, Qingrui Yang

**Affiliations:** 1 Department of Rheumatology and Immunology, Shandong Provincial Hospital Affiliated to Shandong First Medical University, Jinan, Shandong, China; 2 Department of Rheumatology and Immunology, Shandong Provincial Hospital, Cheeloo College of Medicine, Shandong University, Jinan, Shandong, China; 3 Department of Radiology, Shandong Provincial Hospital Affiliated to Shandong First Medical University, Jinan, Shandong, China; 4 Department of Respiratory and Critical Care Medicine, Shandong Provincial Third Hospital, Shandong University, Jinan, China; 5 School of Pharmacy, University College London, London, United Kingdom

**Keywords:** antisynthetase syndrome, interstitial lung disease, cluster analysis, high-resolution computed tomography, Warrick score

## Abstract

**Objective:**

Interstitial lung disease (ILD) is a common and serious complication of anti-synthase syndrome (ASS), exhibiting high heterogeneity. This study aimed to stratify patients with ASS-associated ILD (ASS-ILD) based on their clinical features, high-resolution computed tomography (HRCT) findings, and specific antibody profiles. We also aimed to build a predictive model for severe ASS-ILD to help identify it more accurately.

**Methods:**

We retrospectively analyzed the clinical and laboratory data of 100 patients with ASS-ILD. Unsupervised clustering of clinical characteristics was done using multiple correspondence analysis (MCA) and hierarchical cluster analysis. Univariate and multivariate logistic regression were used to find independent risk factors for severe ASS-ILD, and a risk prediction model was built.

**Results:**

We identified three patient groups: Cluster 1 (*n* = 46), the joint-predominant group, featured joint pain as the primary clinical manifestation. HRCT typically showed a non-specific interstitial pneumonia (NSIP) pattern, anti-Jo-1 antibodies were most common, and lung damage was relatively mild. Cluster 2 (*n* = 41), the fever and Raynaud’s phenomenon group, had significantly higher anti-EJ antibody levels than the other groups. Cluster 3 (*n* = 13), the severe lung group, had much higher Warrick scores than the others. This group also had a higher frequency of a usual interstitial pneumonia (UIP) pattern on HRCT, more cases positive for anti-PL-7 or anti-PL-12 antibodies, more severe lung diffusion problems, and more heart involvement. We created and tested a combined biomarker index based on complement C3 level, rash, and age. This index demonstrated superior performance in distinguishing severe from non-severe ILD in ASS patients, independent of clinical variables such as gender, age, and smoking history.

**Conclusion:**

Identifying ASS-ILD heterogeneity requires comprehensive consideration of HRCT features, antibody profiles, and clinical manifestations. The combined biomarker index (based on complement C3, rash, and age) offers an objective tool for ASS-ILD diagnosis and risk stratification.

## 1 Introduction

Anti-synthase syndrome (ASS) is a rare autoimmune disorder characterized by the presence of anti-aminoacyl-tRNA synthetase (ARS) antibodies in serum, classified within the spectrum of idiopathic inflammatory myopathies (IIM) (1, 2). Its clinical presentation exhibits extreme heterogeneity. The classic “triad” of symptoms includes myositis, arthritis, and interstitial lung disease (ILD) (3). It is also often accompanied by Raynaud’s phenomenon, mechanic’s hands, and other symptoms (3). However, patients frequently present with incomplete forms of the syndrome (3). ILD is the most common manifestation outside the muscles in ASS, occurring in 67 to 100% of patients (4).

ASS shows major differences in how it appears clinically, on imaging scans, and in how it progresses. This creates significant challenges for diagnosis and management. Different anti-ARS antibodies (such as anti-Jo-1, anti-PL-7, anti-PL-12, and anti-EJ) are linked to different clinical patterns of ASS (5). For example, research shows that anti-Jo-1 antibodies are associated with more frequent arthritis and myositis (6–8). In contrast, anti-PL-7 or anti-PL-12 antibodies are often linked to isolated and more severe ILD (3, 9, 10). HRCT is the key test for evaluating ILD in ASS. Common HRCT patterns include usual interstitial pneumonia (UIP), non-specific interstitial pneumonia (NSIP), organizing pneumonia (OP), and lymphocytic interstitial pneumonia (LIP) (4, 11). These imaging patterns are closely tied to patient outcomes. NSIP and OP patterns are linked to better prognosis and response to immunosuppressive treatment. Patients with a UIP pattern, however, tend to have a worse prognosis and higher mortality (12). Studies show that the severity of ILD greatly affects a patient’s outlook, leading to significant illness and potential death (6, 13, 14). Currently, the general approach to treating ILD in ASS is based on studies of myositis patients with ILD (5). There is a lack of precise treatment strategies that account for the unique variability of ASS. Therefore, early identification and precise treatment for patients with ASS-ILD are very important.

However, a single antibody type or imaging pattern may be insufficient to fully capture the complexity of the disease. This study aims to conduct a combined analysis by integrating clinical manifestations, laboratory indicators, antibody profiles, and HRCT features. We used cluster analysis to identify and describe more homogeneous groups of patients with ASS-ILD. Using HRCT, we assessed the disease stage and quantified the extent of pulmonary fibrosis in patients with ASS-ILD. We then used these data to construct a clinical prediction model for severe interstitial lung disease. This approach aims to help move towards more precise treatment for ASS and allow for earlier prevention of complications.

## 2 Materials and methods

### 2.1 Research participants and ethical review

**Figure 1:**
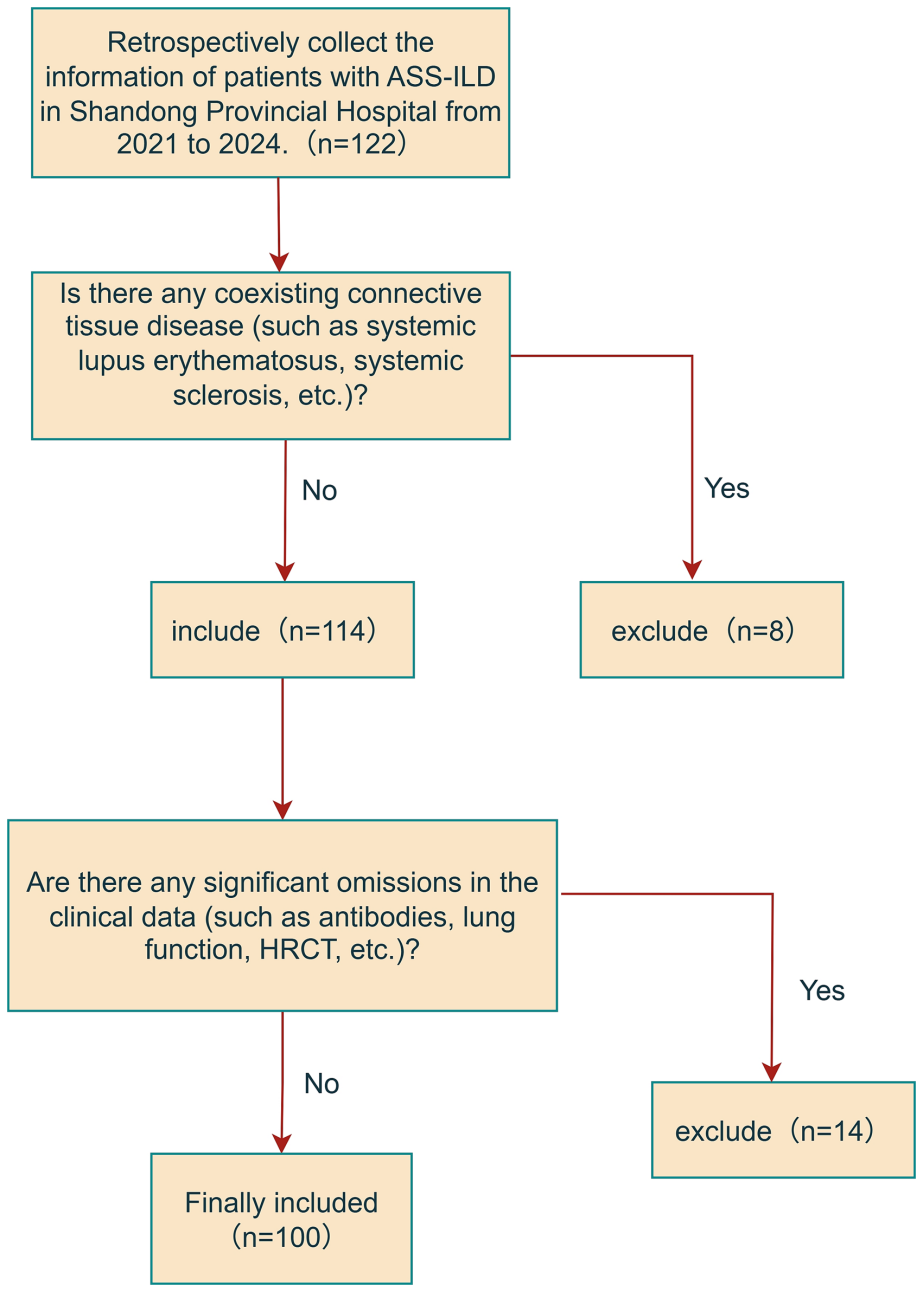
Study cohort.

This study included patients diagnosed with ASS-ILD who were hospitalized between 2021 and 2024 (all from Shandong Provincial Hospital). We retrospectively analyzed their medical records, which included demographic data (gender, age, disease duration, smoking status), clinical characteristics, laboratory results, imaging findings, relevant antibody profiles (anti-synthetase antibodies, anti-Ro52), and pulmonary function tests. The inclusion criteria were as follows: (1) meeting Solomon’s and Connor’s diagnostic criteria (15, 16), with confirmation by at least two rheumatologists; (2) presence of interstitial lung disease (ILD) during the clinical course (17), defined as: (a) chest high-resolution computed tomography (HRCT) showing any of the following (after excluding respiratory infection): ground-glass opacities/reticular opacities, bronchiectasis, interlobular septal/subpleural thickening, honeycombing, or consolidations/cysts; and (b) ILD diagnosis confirmed by the attending rheumatologist or pulmonologist; (3) completion of pulmonary function tests (PFTs) and laboratory tests within 30 days before or after the CT scan. Patients with anti-synthetase syndrome who had other concurrent rheumatic diseases were excluded to minimize the influence of overlapping syndromes. The detailed selection process of the study population is shown in Figure 1. This study was conducted in accordance with the principles of the Declaration of Helsinki and was approved by the Ethics Committee of Shandong Provincial Hospital Affiliated to Shandong First Medical University. Informed consent was obtained from all participants.

### 2.2 Clinical data mining

#### 2.2.1 Variable definition

Cardiac involvement is defined as abnormalities on baseline electrocardiogram (ECG), Holter ECG, transthoracic echocardiography (TTE), or cardiac magnetic resonance imaging (CMR), or elevated levels of cardiac biomarkers such as troponin and B-type natriuretic peptide (18). Arthritis is defined as redness, swelling, warmth, pain, and limited mobility in joints, with or without joint effusion (4). Mechanic’s hands refers to characteristic roughness and scaling of the skin on the hands, particularly the palmar surfaces and fingertips, resembling the hands of a manual laborer. Rash includes the following manifestations: the heliotrope rash (a purplish-red, edematous rash around the eyelids), Gottron’s papules (flat, purplish-red papules over the knuckles and metacarpophalangeal joints), the V-sign (a rash in the V-shaped area of the neck and upper chest), the shawl sign (a rash over the shoulders and upper back), and periungual changes (such as periungual erythema and capillary abnormalities) (4). Fever is defined as an axillary temperature of ≥38.0 °C, confirmed by thermometer measurement, after excluding non-infectious causes such as environmental factors or drug reactions. Raynaud’s phenomenon is defined as episodic vasospasm of the distal extremities (fingers and toes) triggered by cold exposure or stress, which is characterized by sequential blanching, cyanosis, and subsequent reddening (19).

#### 2.2.2 CT examination

CT imaging was performed using a GE Lightspeed VCT XT 64-slice spiral CT scanner. All scans were acquired with patients in the supine position at maximal end-inspiration, covering the lung field from bases to apices. The scan parameters were as follows: tube voltage, 120 kV; tube current, 200 mA; slice thickness, 1 mm; detector configuration, 64 × 0.625 mm; pitch, 0.984:1. Images were reconstructed using both a high-resolution lung algorithm (window level, −600 HU; width, 1,200 HU) and a soft-tissue algorithm (window level, 40 HU; width, 300 HU).

#### 2.2.3 CT assessment and pattern scoring

The primary HRCT pattern for each patient was evaluated by a radiologist and categorized as follows: 1. UIP: Characterized by honeycombing (predominantly subpleural/basal), reticular opacities, and traction bronchiectasis, without significant ground-glass opacities. 2. NSIP: Characterized by ground-glass opacities with fine reticular shadows, uniform distribution, absence of honeycombing, and possible mild bronchiectasis. 3. OP: Characterized by patchy consolidations (with a peribronchovascular or subpleural distribution), possibly featuring a reversed halo sign, and absence of honeycombing. 4. LIP: Characterized by diffuse ground-glass opacities, centrilobular nodules, thin-walled cysts, and lymphadenopathy, without fibrosis. The extent of pulmonary severity was quantified using the Warrick score. A specialist radiologist scored the scan based on specific features: ground-glass opacity (1 point), pleural margin irregularity (2 points), interlobular and subpleural lines (3 points), honeycombing (4 points), and subpleural cysts (5 points). The disease extent was scored based on the number of involved bronchopulmonary segments (out of 18): involvement of 4–9 segments scored 2 points, and involvement of >9 segments scored 3 points. The total Warrick score (range 0–30) was the sum of the feature score and the extent score. All HRCT patterns and Warrick score parameters were assessed independently by two radiologists. Any disagreement was resolved by a third radiologist who was blinded to the study. The Warrick score ≥ 20 was used as the threshold for severe ILD based on the following considerations: (1) The upper quartile of the score in this cohort was 19.75; (2) This threshold was significantly associated with severe diffusing capacity impairment, UIP pattern, and elevated KL-6 (Table 1); (3) Research shows that although the Warrick score thresholds vary among different CTD-ILD subtypes, they can all be used to assess the severity of ILD and functional impairment (20, 21). However, there is no unified standard for ASS-ILD. Therefore, considering the distribution of the cohort and the consensus of three radiologists and two rheumatologists, a score of ≥ 20 was chosen to define severe ILD.

**Table 1:** Characteristics of the ASS-ILD patients in the different HRCT feature.

Variable	All patients (*n* = 100)	NSIP (*n* = 58)	UIP (*n* = 5)	LIP (*n* = 18)	OP (19)	*p*
M/F (n/n)	22/78	12/46	2/3	4/14	4/15	0.789
Age (years)	53.85 ± 11.17	51.36 ± 11.01	53.80 ± 15.29	60.39 ± 7.66	55.26 ± 11.40	0.188
Disease duration	12 (2.00, 24.00)	12.00 (3.00, 24.00)	2.00 (0.50, 7.00)	12.50 (3.00, 21.00)	5.00 (1.15, 4.00)	0.362
Severe pulmonary ventilatory dysfunction (*n*,%)	13 (13%)	5 (8.6%)	2 (40%)	2 (11.1%)	4 (19%)	0.116
Severe pulmonary diffusion dysfunction (*n*,%)	9 (9%)	4 (6.9%)	1 (20%)	1 (5.6%)	3 (15.8%)	0.403
Myosalgia/amyasthenia (*n*,%)	41 (41%)	23 (39.7%)	1 (20%)	12 (66.7%)	5 (26.3%)	0.060
Arthritis (*n*,%)	47 (47%)	24 (41.4%)	2 (40%)	9 (50%)	12 (63.2)	0.387
Fever (*n*,%)	39 (39%)	26 (44.8%)	1 (20%)	5 (27.8%)	7 (36.8%)	0.510
Raynaud’s phenomenon (*n*,%)	9 (9%)	7 (12.1%)	1 (20%)	1 (5.6%)	0 (0%)	0.246
Rash (*n*,%)	26 (26%)	16 (27.6%)	0 (0%)	5 (27.8%)	5 (26.3)	0.750
Cardiac involvement (*n*,%)	32 (32%)	18 (31%)	2 (40%)	6 (33.3%)	6 (31.6%)	0.983
Pleural effusion (*n*,%)	12 (12%)	4 (6.9%)	1 (20%)	2 (11.1%)	5 (26.3%)	0.098
Enlarged lymph nodes (*n*,%)	29 (29%)	16 (27.6%)	2 (40%)	5 (27.8%)	6 (31.6%)	0.899
Peripheral nerve damage	9 (9%)	4 (6.9)				
Anti-JO1+ (*n*,%)	48 (48%)	26 (44.8%)	0 (0%)	8 (44.4%)	14 (73.7%)	0.016*
Anti-PL-7+ (*n*,%)	24 (24%)	13 (22.4%)	5 (100%)	4 (22.2%)	2 (10.5%)	0.002*
Anti-PL-12+ (*n*,%)	8 (8%)	5 (8.6%)	0 (0%)	1 (5.6%)	2 (10.5%)	1.000
Anti-EJ+ (*n*,%)	18 (18%)	14 (24.1)	0 (0%)	3 (16.7%)	1 (5.3%)	0.250
Anti-OJ+ (*n*,%)	1 (1%)	0 (0%)	0 (0%)	1 (5.6%)	0 (0%)	0.230
Anti-Ha+ (*n*,%)	1 (1%)	0 (0%)	0 (0%)	1 (5.6%)	0 (0%)	0.230
Warrick	15.00 (15.00, 19.75)	15.00 (15.00, 18.00)	23.00 (18.00, 30.00)	15.00 (15.00, 23.00)	15.00 (15.00, 23.00)	0.026*
RBC (10^12/L)	4.33 (4.08, 4.71)	4.36 (4.08, 4.79)	4.53 (4.20, 4.77)	4.16 (4.08, 4.43)	4.45 (4.14, 4.82)	0.310
WBC (10^9/L)	8.51 (6.33, 12.05)	8.18 (6.30, 11.20)	10.60 (8.22, 11.26)	8.77 (4.62, 12.41)	10.14 (6.46, 12.43)	0.502
PLT (10^9/L)	274.68 ± 94.85	277.40 ± 95.38	256.40 ± 67.67	293.00 ± 84.68	253.84 ± 109.43	0.371
HB (g/L)	125.79 ± 16.54	125.97 ± 16.73	132.40 ± 18.81	117.17 ± 15.16	131.68 ± 14.11	0.184
CRP (mg/ml)	7.43 (2.13, 17.61)	6.83 (2.30, 18.60)	12.10 (5.40, 16.38)	7.15 (2.10, 16.94)	9.10 (2.07, 17.07)	0.995
ESR (mm/h)	23.00 (12.25, 37.25)	22.50 (14.00, 32.00)	29.00 (27.00, 35.00)	43.00 (21.00, 65.00)	14.00 (9.50, 20.50)	0.006*
IgG (g/L)	14.45 (11.10, 17.03)	14.50 (12.00, 17.10)	15.10 (11.10, 16.80)	14.40 (12.20, 18.00)	11.90 (9.49, 15.50)	0.499
IgM (g/L)	1.40 (0.89, 1.73)	1.44 (1.01, 1.75)	1.03 (0.83, 1.38)	1.31 (0.83, 1.64)	1.55 (0.88, 1.81)	0.546
IgA (g/L)	2.49 (1.92, 3.06)	2.60 (2.02, 3.54)	2.39 (2.39, 2.72)	2.81 (2.29, 3.39)	1.73 (1.28, 2.14)	0.002*
C3 (g/L)	1.07 (0.94, 1.23)	1.05 (0.95, 1.21)	1.10 (1.02, 1.15)	1.12 (1.00, 1.30)	1.02 (0.94, 1.20)	0.529
C4 (g/L)	0.25 (0.18, 0.29)	0.25 (0.19, 0.30)	0.26 (0.25, 0.32)	0.22 (0.15, 0.30)	0.25 (0.17, 0.28)	0.552
ALB (g/L)	34.70 (31.00, 38.50)	35.00 (31.50, 38.60)	32.90 (31.00, 36.90)	35.55 (32.00, 39.00)	31.80 (28.25, 36.05)	0.178
GLO (g/L)	30.95 (27.70, 34.80)	31.55 (28.70, 34.80)	31.10 (25.00, 34.40)	32.30 (28.10 37.10)	26.40 (24.70, 30.85)	0.048*

M/F: Male/Female, Anti-JO1: Anti-histidyl-tRNA synthetase antibody, Anti-PL-7: Anti-threonyl-tRNA synthetase antibody, Anti-PL-12: Anti-alanyl-tRNA synthetase antibody, Anti-EJ: Anti-glycyl-tRNA synthetase antibody, Anti-OJ: Anti-isoleucyl-tRNA synthetase antibody, Anti-Ha: Anti-tyrosyl-tRNA synthetase antibody, RBC: Red Blood Cell count, WBC: White Blood Cell count, PLT: Platelet count, HB: Hemoglobin, CRP: C-Reactive Protein, ESR: Erythrocyte Sedimentation Rate, C3: Complement component 3, C4: Complement component 4, ALB: Albumin, GLO: Globulin, IgA/M/G: immunoglobulin A/M/G, UIP: Usual interstitial pneumonia, NSIP: Nonspecific interstitial pneumonia, OP: Organizing Pneumonia, LIP: lymphocytic interstitial pneumonia, Characteristics were compared among the group using the one-way ANOVA, Kruskal-Wallis test or Chi-square test as appropriate, *p < 0.05.

### 2.3 Statistical analysis

**Figure 2:**
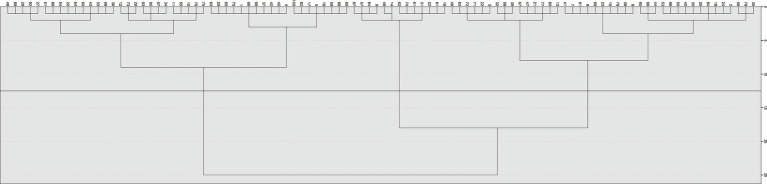
The results of the hierarchical cluster analysis are displayed in the dendrogram.

The data were analyzed using IBM SPSS Statistics (version 26.0). Quantitative data are presented as mean ± standard deviation (for normally or approximately normally distributed data) or median with interquartile range (for non-normally distributed data). Qualitative data are expressed as frequency and percentage. For comparisons between groups, statistical tests including the Chi-square test, t-test, Mann–Whitney U test, one-way ANOVA, and Kruskal-Wallis test were applied as appropriate. Risk factors for severe ILD were identified using logistic regression analysis, with optimal cut-off values determined by receiver operating characteristic (ROC) curves. Patient subtypes were derived through Multiple Correspondence Analysis (MCA) and hierarchical cluster analysis. The 23 variables included in MCA were selected based on the following three criteria: (1) they have been proven to have a definite clinical correlation with ASS-ILD in previous literature (3–5); (2) the data integrity is good (the missing rate is <5%) in all patients; (3) they comprehensively cover the core dimensions of the ASS-ILD phenotype, including demographic characteristics, clinical manifestations, antibody profiles, HRCT patterns, and functional impairment. The number of clusters was selected based on the hierarchical changes observed in the dendrogram (Figure 2) and clinical relevance. One-way ANOVA confirmed significant differences among the three subtypes across both MCA dimensions (*p* < 0.001). The robustness of the clustering results was validated using K-means clustering, which showed high consistency with the systematic clustering results (Kappa = 0.856).

## 3 Result

### 3.1 Demographic characteristics, clinical manifestations, and primary organ involvement

This study included 100 patients with ASS-ILD. The mean age at disease onset was 53.85 ± 11.17 years, and the median disease duration was 12 months (interquartile range, 2–24 months). The cohort consisted of 22 males and 78 females. Muscle involvement and joint involvement were the most common clinical features, present in 41 and 47% of patients, respectively. Other observed manifestations included fever (39%), cardiac involvement (32%), lymphadenopathy (29%), rash (26%), and Raynaud’s phenomenon (9%). The median Warrick score was 15. Among the 100 patients, anti-Jo-1 antibody was the most frequently detected (48%), followed by anti-PL-7 (24%), anti-EJ (18%), and anti-PL-12 (8%). In this study, NSIP (*n* = 58, 58%) was the most common HRCT finding among ASS ILD patients. Anti-JO-1 antibody positivity was significantly higher in the OP group (73.7%) compared to other groups (*p* = 0.016). The LIP group showed the highest erythrocyte sedimentation rate (ESR) and serum IgA concentration. Patients with a UIP pattern had the highest rate of anti-PL-7 antibody positivity (100%, *p* = 0.002) and the highest Warrick score (*p* = 0.026). Detailed data are presented in Table 1.

### 3.2 Identification of clinical subtypes in ASS-ILD through multivariable cluster analysis

**Figure 3:**
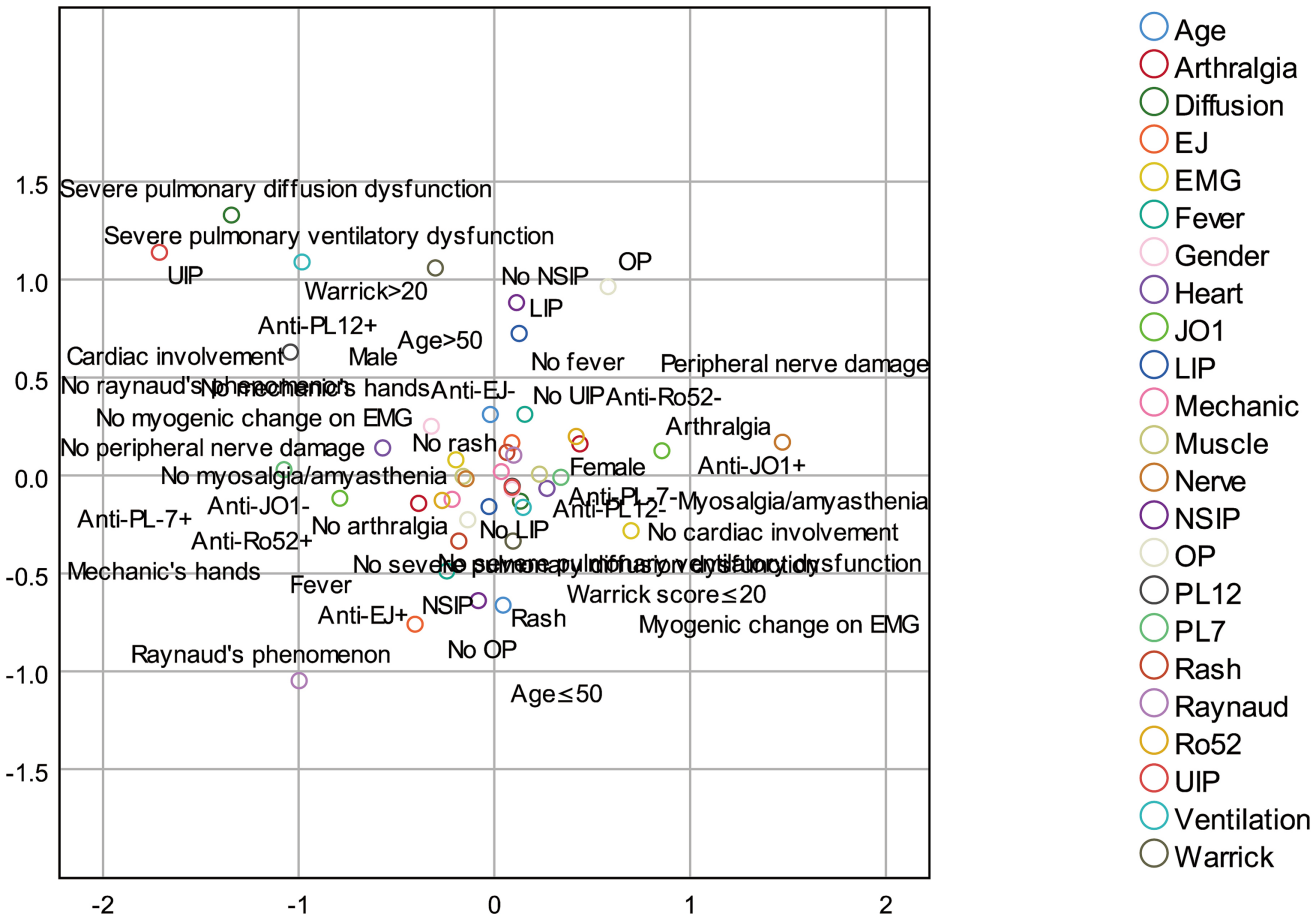
Multiple correspondence analysis (MCA). The variables included in this analysis were: age, gender, arthritis, myalgia/muscle weakness, electromyography showing myopathic damage, skin rash, Raynaud’s phenomenon, fever, peripheral nerve damage, cardiac involvement, pleural effusion, pulmonary diffusion function, pulmonary ventilation function, HRCT findings (including NSIP, LIP, OP, UIP), anti-synthetase antibodies (anti-JO-1, anti-PL-12, anti-PL-7, anti-EJ), anti-Ro52 antibody, and the Warrick score.

This study performed multiple correspondence analysis (MCA) based on 23 variables: age, sex, arthritis, myalgia/muscle weakness, myopathic changes on electromyography, rash, Raynaud’s phenomenon, fever, peripheral neuropathy, cardiac involvement, pleural effusion, pulmonary diffusion capacity, pulmonary ventilation function, HRCT pattern (NSIP, LIP, OP, UIP), type of anti-synthetase antibody (anti-Jo-1, anti-PL-12, anti-PL-7, anti-EJ), anti-Ro52 status, and Warrick score. The results of MCA show that the eigenvalues of the first two dimensions are 2.78 and 2.46 respectively, both greater than 1, indicating that these two dimensions can effectively capture the main variations of the original 23 variables. The Cronbach’s alpha coefficients of the two dimensions are 0.67 and 0.62 respectively, indicating that the dimensional structure is stable and reliable. The two-dimensional MCA plot (Figure 3) displays associations between these variables. Two points located close together indicate that the corresponding features frequently co-occurred in patients. Warrick score <20 showed closely associations with Anti-Jo-1 positivity, anti-EJ positivity, Raynaud’s phenomenon, rash, fever, arthritis, myalgia, myopathic changes on electromyography and NSIP. Warrick score >20 was closely linked to anti-PL-12 positivity, severe impairment in both pulmonary diffusion and ventilation, and UIP. MCA transformed the 23 categorical variables into continuous factor coordinates. Hierarchical cluster analysis was then performed on the two principal components derived from MCA, using Euclidean distance and Ward’s linkage method. The resulting dendrogram (Figure 2) clearly illustrates the clustering process and serves as an intuitive tool for determining the optimal number of clusters. The vertical axis represents distance, and the horizontal axis represents individual patients. A long vertical branch indicates the merging of two distinct clusters. By drawing a vertical line above a long branch, the number of horizontal branches it intersects suggests the suitable number of clusters. Based on this systematic cluster analysis, the 100 ASS-ILD patients were classified into three distinct clinical subtypes. The characteristics of the three clusters are summarized in Table 2. To evaluate the robustness of clustering, we calculated the average silhouette coefficient of the three-cluster scheme, and the result was 0.61, suggesting that the clustering structure is reasonable. Furthermore, the K-means clustering method was used to verify the hierarchical clustering results. The Kappa value of the consistency between the results of the two clustering methods was 0.86, indicating that the clustering results have good robustness.

**Table 2:** Comparison of clinical features and laboratory indicators across the three clusters.

Variable	All patents (*n* = 100)	Cluster 1 (*n* = 46)	Cluster 2 (*n* = 41)	Cluster 3 (*n* = 13)	*p*
Gender (M/F)	22/78	8/38	9/32	5/8	0.266
Age (years)	53.85 ± 11.17	54.54 ± 10.26	50.49 ± 11.89	62.00 ± 7.09	0.017*
Disease duration (months)	11.50 (2.00, 24.00)	12.00 (2.00, 41.00)	10.00 (3.00, 18.00)	2.00 (1.00, 36.00)	0.481
Warrick score	16.67 ± 4.70	16.35 ± 4.79	15.24 ± 3.08	22.23 ± 4.85	0.000**
Warrick score>20 (*n*,%)	24 (24%)	11 (23.9%)	3 (7.3%)	10 (76.9%)	0.000**
UIP (*n*,%)	5 (5%)	0 (0%)	1 (2.4%)	4 (30.8%)	0.000**
NSIP (*n*,%)	58 (58%)	21 (45.7%)	35 (85.4%)	2 (15.4%)	0.000**
LIP (*n*,%)	18 (18%)	10 (21.7%)	4 (9.8%)	4 (30.8%)	0.153
OP (*n*,%)	19 (19%)	15 (32.6%)	1 (2.4%)	3 (23.1%)	0.002*
Severe pulmonary ventilatory dysfunction (*n*,%)	13 (13%)	2 (4.3%)	3 (7.3%)	8 (61.5%)	0.000*8
Severe pulmonary diffusion dysfunction (*n*,%)	9 (9%)	0 (0%)	2 (4.9%)	7 (53.8%)	0.000**
Myosalgia/amyasthenia (*n*,%)	41 (41%)	22 (47.8%)	14 (34.1%)	5 (38.5%)	0.424
Arthritis (*n*,%)	47 (47%)	32 (69.6%)	9 (22%)	6 (46.2%)	0.000**
Fever (*n*,%)	39 (39%)	12 (26.1%)	24 (58.5%)	3 (23.1%)	0.004*
Raynaud’s phenomenon (*n*,%)	9 (9%)	0 (0%)	9 (22%)	0 (0%)	0.001*
Myogenic change on EMG (*n*,%)	22 (22%)	14 (30.4%)	7 (17.1%)	1 (7.7%)	0.133
Rash (*n*,%)	26 (26%)	9 (19.6%)	15 (36.6%)	2 (15.4%)	0.126
Cardiac involvement (*n*,%)	32 (32%)	8 (17.4%)	16 (39%)	8 (61.5%)	0.005*
Pleural effusion (*n*,%)	12 (12%)	5 (10.9%)	5 (12.2%)	2 (15.4%)	0.913
Peripheral nerve damage (*n*,%)	9 (9%)	9 (19.6%)	0 (0%)	0 (0%)	0.003*
Anti-JO1+ (*n*,%)	48 (48%)	42 (91.3%)	5 (12.2%)	1 (7.7%)	0.000**
Anti-PL-7+ (*n*,%)	24 (24%)	0 (0%)	17 (41.5%)	7 (53.8%)	0.000**
Anti-PL-12+ (*n*,%)	8 (8%)	0 (0%)	4 (9.8%)	4 (30.8%)	0.001*
Anti-EJ+ (*n*,%)	18 (18%)	3 (6.5%)	14 (34.1%)	1 (7.7%)	0.002*
Anti-HA+ (*n*,%)	1 (1%)	1 (2.2%)	0 (0%)	0 (0%)	1.000
Anti-OJ+ (*n*,%)	1 (1%)	0 (0%)	1 (2.4%)	0 (0%)	0.483
Anti-Ro52+ (*n*,%)	61 (61%)	21 (45.7%)	30 (73.2%)	10 (76.9%)	0.014*
RBC (10^12/L)	4.33 (4.09, 4.70)	4.33 (4.12, 4.62)	4.31 (4.09, 4.83)	4.38 (3.69, 4.62)	0.708
WBC (10^9/L)	8.51 (6.35, 12.02)	8.64 (6.32, 12.32)	8.09 (6.19, 10.24)	10.60 (8.22, 12.07)	0.389
PLT (10^9/L)	274.68 ± 94.85	281.93 ± 96.34	272.15 ± 99.75	257.00 ± 75.46	0.394
HB (g/L)	125.79 ± 16.54	126.59 ± 14.94	126.42 ± 17.17	121.00 ± 20.17	0.276
CRP (mg/ml)	7.43 (2.15, 17.48)	8.31 (3.00, 17.75)	5.00 (2.00, 22.69)	11.70 (2.93, 16.38)	0.678
ESR (mm/h)	23.00 (12.50, 36.50)	17.50 (10.00, 34.00)	24.00 (17.00, 34.00)	29.00 (12.00, 38.00)	0.455
BUN (mg/dL)	5.30 (4.13, 7.48)	5.50 (4.10, 7.60)	4.60 (3.90, 6.20)	6.20 (5.30, 9.70)	0.015*
Cr (mg/dL)	50.40 (42.45, 58.68)	52.35 (46.20, 59.20)	49.90 (41.20, 55.80)	50.10 (40.67, 61.00)	0.323
IgG (g/L)	14.45 (11.10, 17.03)	14.50 (10.50, 16.30)	14.50 (12.20, 17.20)	13.30 (11.10, 15.10)	0.484
IgA (g/L)	2.49 (1.92, 3.06)	2.28 (1.82, 3.06)	2.60 (2.28, 3.16)	1.97 (1.73, 2.72)	0.155
IgM (g/L)	1.40 (0.89, 1.73)	1.57 (1.50, 1.93)	1.36 (0.88, 1.70)	0.91 (0.83, 1.38)	0.051
C3 (g/L)	1.065 (0.95, 1.23)	1.04 (0.93, 1.16)	1.10 (0.99, 1.27)	1.07 (0.94, 1.20)	0.305
C4 (g/L)	0.25 (0.18, 0.29)	0.23 (0.18, 0.27)	0.25 (0.17, 0.30)	0.27 (0.21, 0.31)	0.326
KL-6 (U/ml)	1574.20 ± 1071.13	1185.13 ± 443.00	1733.93 ± 1073.68	2447.15 ± 1842.25	0.000**

M/F: Male/Female, UIP: Usual interstitial pneumonia, NSIP: Nonspecific interstitial pneumonia, OP: Organizing Pneumonia, LIP: lymphocytic interstitial pneumonia, Anti-JO1: Anti-histidyl-tRNA synthetase antibody, Anti-PL-7: Anti-threonyl-tRNA synthetase antibody, Anti-PL-12: Anti-alanyl-tRNA synthetase antibody, Anti-EJ: Anti-glycyl-tRNA synthetase antibody, Anti-OJ: Anti-isoleucyl-tRNA synthetase antibody, Anti-Ha: Anti-tyrosyl-tRNA synthetase antibody, RBC: Red Blood Cell count, WBC: White Blood Cell count, PLT: Platelet count, HB: Hemoglobin, CRP: C-Reactive Protein, ESR: Erythrocyte Sedimentation Rate, BUN: Blood Urea Nitrogen, Cr: Creatinine, C3: Complement component 3, C4: Complement component 4, IgA/M/G: immunoglobulin A/M/G, KL-6: Krebs von den Lungen-6, Characteristics were compared among the group using the one-way ANOVA, Kruskal-Wallis test or Chi-square test as appropriate. *p < 0.05, **p < 0.001.

### 3.3 Cluster 1: arthralgia-predominant with mild pulmonary involvement and anti-jo-1 antibody dominance

Patients in Cluster 1 had a mean disease duration of 12 months, a mean onset age of 54.54 years, a mean Warrick score of 16.35, and a mean KL-6 level of 1185.13 U/mL. The primary HRCT pattern was NSIP (45.7%), followed by OP (32.6%) and LIP (21.7%); no UIP pattern was observed. Regarding antibody profiles, anti-Jo-1 positivity was predominant (91.3%), while a small proportion of patients were positive for anti-EJ (6.5%) or anti-HA (2.2%). No patients tested positive for anti-PL-7, anti-PL-12, or anti-OJ antibodies. Clinically, this cluster was characterized by a higher prevalence of arthralgia (69.6%) and peripheral neuropathy (19.6%) compared to the other clusters. Notably, no patients in this group exhibited severe pulmonary diffusion impairment, and KL-6 levels were the lowest among all clusters, indicating relatively milder lung involvement.

### 3.4 Cluster 2: NSIP with fever and Raynaud’s phenomenon as a distinct subtype

Patients in Cluster 2 had a mean disease duration of 10 months, a mean onset age of 50.49 years, a mean Warrick score of 15.24, and a mean KL-6 level of 1733.93 U/mL. On HRCT, the NSIP pattern was predominant (85.4%), with LIP, OP, and UIP observed in 9.8, 2.4, and 2.4% of patients, respectively. Antibody profiles were diverse: anti-PL-7 was most common (41.5%), followed by anti-EJ (34.1%), anti-Jo-1 (12.2%), anti-PL-12 (9.8%), and anti-OJ (2.4%). No anti-HA positivity was detected. Clinically, this cluster was distinguished by a significantly higher prevalence of fever (58.5%) and Raynaud’s phenomenon (22.0%) compared to the other groups.

### 3.5 Cluster 3: severe pulmonary involvement with UIP pattern and anti-PL-7/PL-12 positivity

Patients in Cluster 3 had a mean disease duration of 2 months, a mean age at onset of 62 years, a mean Warrick score of 22.23, and a mean KL-6 level of 2447.15 U/mL. The HRCT patterns were variable: NSIP (15.4%), LIP (30.8%), OP (23.1%), and UIP (30.8%). Antibody profiles were dominated by anti-PL-7 (53.8%) and anti-PL-12 (30.8%); anti-Jo-1 and anti-EJ were each positive in 7.7% of patients. No anti-OJ or anti-HA antibodies were detected. Compared with Clusters 1 and 2, Cluster 3 patients were older, had a shorter disease duration, a higher frequency of the UIP pattern, the highest Warrick score, the highest rates of anti-PL-7 and anti-PL-12 positivity, a greater proportion of cardiac involvement (61.5%), and the highest KL-6 levels. These features collectively indicate more severe pulmonary injury, greater overall disease severity, and a potentially poorer prognosis in this cluster.

### 3.6 A composite index comprising rash, age, and complement C3 predicts mild and severe ILD

**Figure 4:**
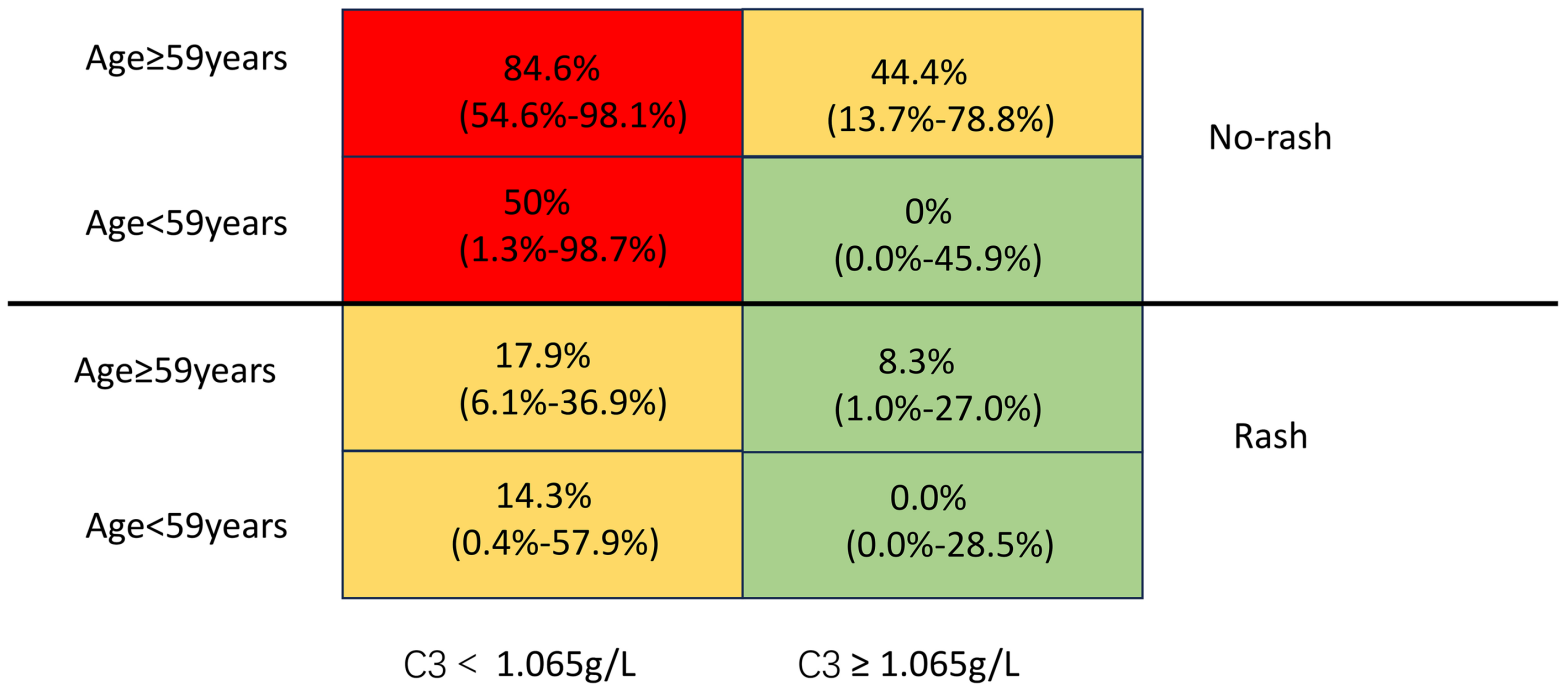
Matrix prediction model.

**Figure 5:**
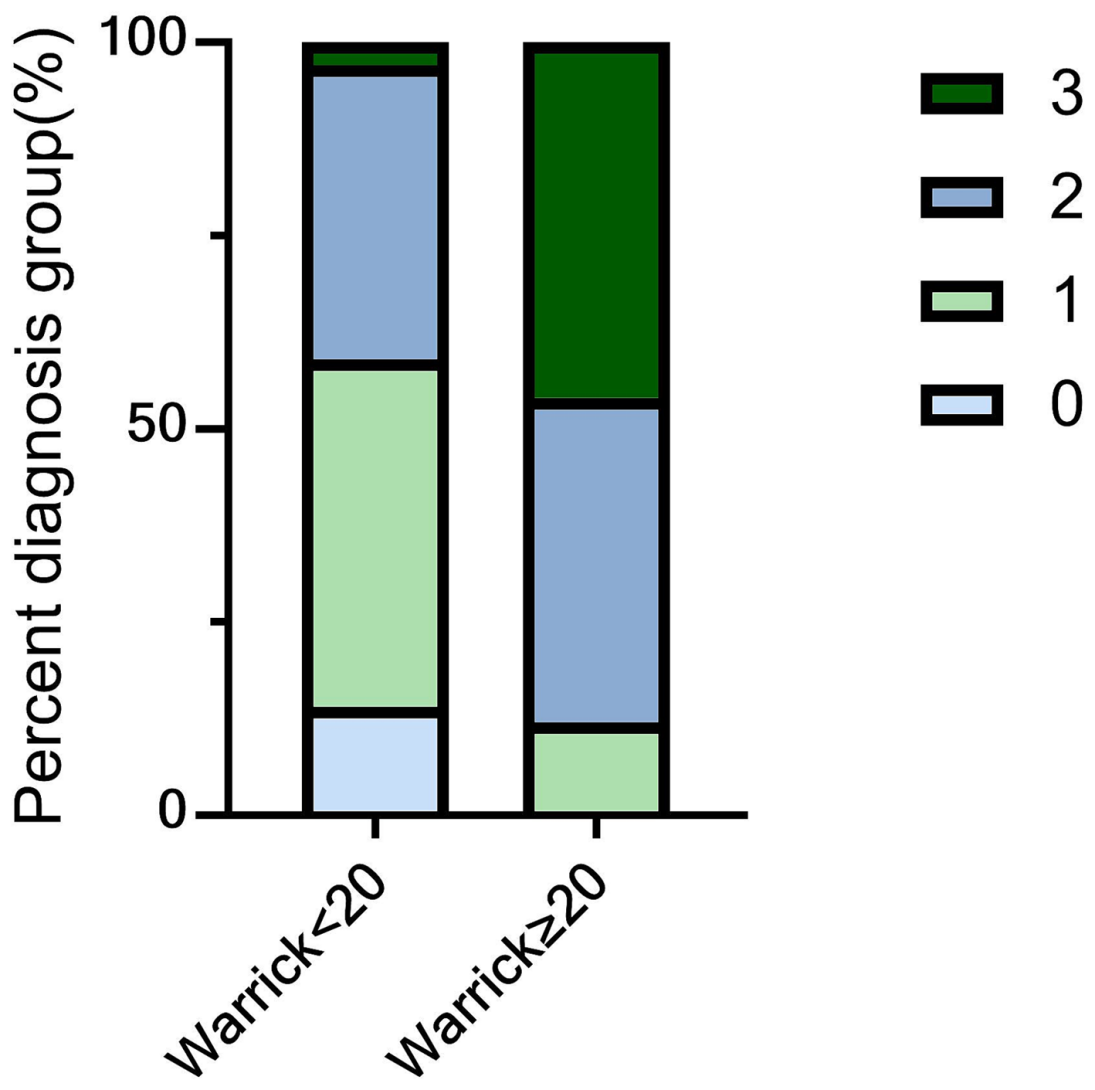
The biomarker index score (0–3) was distributed across patient groups in the cohort.

Severe ASS-ILD was defined by a Warrick score ≥20. To evaluate the predictive ability of the Warrick score for lung function impairment, ROC analysis was performed with the Warrick score as the test variable and severe ventilation function impairment as the state variable. The obtained AUC was 0.751 (95% CI: 0.582–0.920, *p* = 0.013), indicating that the Warrick score has a moderate predictive value for ventilation function impairment. According to the principle of maximizing the Youden index, the optimal cut-off value was determined to be 21.5 points, with a corresponding sensitivity of 66.7% and a specificity of 80.2%. Patients with severe ILD were older (*p* < 0.001), had a higher proportion of males (*p* = 0.035), and a higher proportion with severe pulmonary diffusion impairment (*p* = 0.006). In contrast, rash (*p* = 0.024) and fever (*p* = 0.036) were less common compared to the non-severe group. While NSIP was the most common HRCT pattern in both groups, its frequency was significantly lower in the severe group (*p* = 0.020). Compared with the non-severe group, the severe group showed significantly elevated serum creatinine (*p* = 0.005) and blood urea nitrogen (*p* = 0.003), but lower red blood cell counts (*p* = 0.032) and complement C3 levels (*p* = 0.010). Detailed comparisons are presented in Table 3. To identify risk factors for severe ASS-ILD, we constructed a clinical prediction model using logistic regression analysis. Variables with *p* < 0.05 in univariate analysis were included in the multivariate model, which incorporated age, sex, NSIP pattern, fever, rash, and serum C3 concentration. The results identified age as an independent risk predictor for a Warrick score ≥20, with each additional year increasing the risk by 1.11-fold. Conversely, rash and higher serum C3 levels serve as protective factors. Each unit increase in C3 concentration is associated with an approximately 96% reduction in the risk of severe ASS-ILD, while the presence of rash is linked to an 83% lower risk of severe disease. ROC analysis indicated that patients older than 59 years, without rash, and with a serum C3 concentration below 1.065 g/L had a higher probability of developing severe ILD (see Tables 4, 5). To visualize these interactions, a matrix model was created combining age (≥59 or <59 years), rash (present or absent), and C3 level (≥1.065 or <1.065 g/L). Each cell represents the relative risk probability of severe ILD, with 95% confidence intervals in parentheses. High-risk (≥50% probability), moderate-risk (10–49%), and low-risk (<10%) cells are color-coded red, yellow, and green, respectively (Figure 4). These three variables—age, rash, and C3—were integrated into a final biomarker index. For each of the 100 ASS-ILD patients, a score (0–3) was calculated based on the number of these biomarkers exceeding their respective thresholds (age ≥59 years, absence of rash, C3 < 1.065 g/L). The distribution of scores across groups is shown in Figure 5. After adjusting for age, sex, and smoking history, a biomarker index score ≥2 was significantly associated with severe ASS-ILD (odds ratio 8.84, 95% CI 2.22–35.13, *p* = 0.002), as detailed in Table 6. To verify the predictive efficacy of the biomarker index for severe ILD, we plotted a ROC curve with the index score (0–3 points) as the test variable and severe ILD (Warrick score ≥ 20) as the state variable. The results showed that the AUC was 0.825 (95% CI: 0.728–0.921, *p* < 0.001), indicating that the index had good discriminatory ability. According to the principle of maximizing the Youden index, the optimal cut-off value was determined to be an index score ≥ 2 points, with a corresponding sensitivity of 87.5% and a specificity of 59.2% (Table 7).

**Table 3:** Characteristics of the ASS-ILD patients in the different Warrick score.

Variable	Warrick<20 (*n* = 76)	Warrick≧20 (*n* = 24)	*p*
M/F (n/n)	13/63	9/15	0.035*
Smoke	7 (9.2)	6 (25)	0.098
Age (years)	53.00 (46.50, 57.00)	62.50 (54.00, 68.50)	0.000**
Disease duration (months)	10.50 (2.00, 22.50)	12.00 (2.00, 43.00)	0.539
UIP (*n*,%)	2 (2.6)	3 (12.5)	0.051
NSIP (*n*,%)	49 (64.5)	9 (37.5)	0.020*
LIP (*n*,%)	12 (15.8)	6 (25)	0.472
OP (*n*,%)	13 (17.1)	6 (25)	0.575
Severe pulmonary ventilatory dysfunction (*n*,%)	8 (10.5)	5 (20.8)	0.337
Severe pulmonary diffusion dysfunction (*n*,%)	3 (3.9)	6 (25)	0.006*
Muscle (*n*,%)	32 (42.1)	9 (37.5)	0.689
Arthritis (*n*,%)	37 (48.7)	10 (41.7)	0.548
Fever (*n*,%)	34 (44.7)	5 (20.8)	0.036*
Raynaud (*n*,%)	9 (11.8)	0 (0)	0.174
Rash (*n*,%)	24 (36.1)	2 (8.3)	0.024*
Mechanic (*n*,%)	12 (15.8)	2 (8.3)	0.562
Cardiac involvement (*n*,%)	24 (36.1)	8 (33.3)	0.872
Effusion (*n*,%)	9 (11.8)	3 (12.5)	0.931
RBC (10^12/L)	4.38 (4.11, 4.81)	4.16 (3.74, 4.55)	0.032*
Anti-JO1+ (*n*,%)	39 (51.3)	9 (37.5)	0.238
Anti-PL-7+ (*n*,%)	17 (22.4)	7 (29.2)	0.497
Anti-PL-12+ (*n*,%)	5 (6.6)	3 (12.5)	0.617
Anti-EJ+ (*n*,%)	14 (18.4)	4 (16.7)	1.000
Anti-HA+ (*n*,%)	0 (0)	1 (4.2)	0.240
Anti-OJ+ (*n*,%)	1 (1.3)	0 (0)	1.000
WBC (10^9/L)	8.40 (6.35, 11.33)	9.18 (6.38, 12.27)	0.692
PLT (10^9/L)	278.88 ± 94.29	261.38 ± 97.41	0.433
CRP (mg/ml)	5.84 (2.15, 16.79)	10.25 (3.67, 23.23)	0.417
ESR (mm/h)	21.50 (13.50, 32.50)	29.00 (12.00, 40.00)	0.230
BUN (mg/dL)	4.85 (4.00, 6.25)	7.00 (5.30, 10.20)	0.003*
Cr (mg/dL)	50.37 ± 9.87	59.36 ± 20.85	0.005*
C3 (g/L)	1.123 ± 0.234	1.000 ± 0.138	0.010*
C4 (g/L)	0.25 (0.19, 0.30)	0.22 (0.18, 0.29)	0.563

M/F: Male/Female, UIP: Usual interstitial pneumonia, NSIP: Nonspecific interstitial pneumonia, OP: Organizing Pneumonia, LIP: lymphocytic interstitial pneumonia, Anti-JO1: Anti-histidyl-tRNA synthetase antibody, Anti-PL-7: Anti-threonyl-tRNA synthetase antibody, Anti-PL-12: Anti-alanyl-tRNA synthetase antibody, Anti-EJ: Anti-glycyl-tRNA synthetase antibody, Anti-OJ: Anti-isoleucyl-tRNA synthetase antibody, Anti-Ha: Anti-tyrosyl-tRNA synthetase antibody, RBC: Red Blood Cell count, WBC: White Blood Cell count, PLT: Platelet count, CRP: C-Reactive Protein, ESR: Erythrocyte Sedimentation Rate, BUN: Blood Urea Nitrogen, Cr: Creatinine, C3: Complement component 3, C4: Complement component 4, Characteristics were compared among the group using the Chi-square test, t-test or Mann–Whitney U as appropriate. *p < 0.05, **p < 00.001.

**Table 4:** Logistic regression analysis of independent risk factors for severe ASS-ILD.

Single factor regression	OR	95%CI	*p*	Multi-factor regression	OR	95%CI	*p*
Female	0.34	(0.12,0.95)	0.040*	Female	0.38	(0.10,1.44)	0.153
UIP	5.29	(0.83,33.74)	0.078				
NSIP	0.33	(0.13,0.86)	0.022*	NSIP	0.38	(0.12,1.26)	0.114
Fever	0.33	(0.11,0.96)	0.042*	Fever	0.39	(0.10,1.47)	0.163
Rash	0.20	(0.04,0.91)	0.037*	Rash	0.17	(0.03,0.99)	0.049*
Age	1.11	(1.05,1.18)	0.000*	Age	1.11	(1.04,1.18)	0.000**
RBC	0.71	(0.32,1.58)	0.400				
BUN	1.00	(0.99,1.01)	0.545				
C3	0.04	(0.003, 0.59)	0.019*	C3	0.04	(0.001,0.83)	0.038*

UIP: Usual interstitial pneumonia, NSIP: Nonspecific interstitial pneumonia, BUN: Blood Urea Nitrogen, C3: Complement component 3, Multivariable analysis was implemented through binary logistic regression models adjusted for potential confounders. *p < 0.05, **p < 0.001.

**Table 5:** Determine the optimal cut-off point from the ROC curve.

Variable	AUC	*p*	Optimal thresholds	Sensitivities%	Specificity%
C3	0.675	0.009*	1.065 g/L	0.75%	0.579%
Age	0.758	0.000**	59 years	0.667%	0.816%

ROC analysis demonstrated good diagnostic performance of C3 (AUC = 0.675, *p* = 0.009) and age (AUC = 0.785, *p* < 0.0001) for severe ASS-ILD, with optimal cut-off values of 1.065 g/L and 59 years, respectively.

**Table 6:** Performance of the index to distinguish severe-ASS-ILD from ASS-ILD.

Variable	B	OR	95%CI	*p*
Index score≥2	2.178	8.84	(2.22,35.13)	0.002*
Gender	−1.067	0.35	(0.06,1.92)	0.225
Smoke	0.88	1.09	(0.14,8.28)	0.932
Age	0.84	1.09	(1.02,1.16)	0.007*

After adjusting for gender, age and smoking history, the biomarker index score remained strongly associated with severe ASS-ILD (biomarker index score ≥ 2; odds ratio [OR] 8.84, 95% confidence interval [CI] 2.22–35.13; *p* = 0.002).

**Table 7:** Determine the optimal cut-off point from the ROC curve.

Variable	AUC	*p*	Optimal thresholds	Sensitivities%	Specificity%
Index score	0.825	0.000**	2 score	0.875%	0.592%

ROC analysis demonstrated good diagnostic performance of index score (AUC = 0.825, 95% confidence interval: 0.728–0.921, *p* < 0.001), with optimal cut-off values of 2 score.

## 4 Discussion

ASS-ILD can develop before clinical symptoms appear. Although HRCT is the gold standard for diagnosing ILD, its findings are not routinely integrated with clinical and laboratory data. Shi et al. (19) previously identified three clinical subtypes in ASS using unsupervised clustering of symptoms and routine lab tests. However, that study omitted imaging and specific anti-synthetase antibody profiles, and did not focus on severe ASS-ILD. Our retrospective analysis of 100 ASS-ILD patients employed unsupervised clustering, successfully identifying three subtypes with distinct clinical, imaging, and serological characteristics. We constructed a predictive model for severe ASS-ILD based on age, rash, and serum complement C3 levels. These findings confirm the high heterogeneity of ASS-ILD and provide a new framework for understanding its mechanisms, supporting early risk stratification and precision treatment.

The three clinical subtypes delineated in this study profoundly reflect the complexity of the ASS-ILD disease spectrum. Cluster 1 is characterized by a very high prevalence of anti-Jo-1 antibodies, prominent articular manifestations, and relatively mild pulmonary involvement. This aligns with prior reports: Hervier et al. demonstrated that anti-Jo-1 positivity correlates with a lower frequency of ILD and a higher frequency of myositis compared to anti-PL-7 and anti-PL-12 positivity (22). Cavagna et al. found that among anti-Jo-1-positive patients, arthritis was the most common clinical manifestation, typically presenting as a symmetrical polyarthritis (3). Pinal-Fernandez et al. indicate that anti-Jo-1 antibodies are associated with more pronounced myositis and articular damage alongside milder ILD (10). Lei et al. (23) similarly identified arthralgia as a protective factor for overall survival when comparing dermatomyositis with ASS-associated ILD. These collective observations support our findings regarding Cluster 1. In contrast, Cluster 3 is defined by advanced age, positivity for anti-PL-7 or anti-PL-12 antibodies, a UIP pattern on imaging, severe impairment of pulmonary diffusion, the highest proportion of anti-Ro-52 co-positivity, and the highest Warrick scores and serum KL-6 levels, signifying the most severe pulmonary phenotype. This finding aligns with recent studies indicating that non-Jo-1 antibodies, particularly anti-PL-7 and anti-PL-12, are strong risk markers for severe and rapidly progressive ILD in ASS (10). Among rheumatoid arthritis-associated interstitial lung disease (RA-ILD) patients, those with the UIP pattern exhibit poorer survival and disease trajectories similar to idiopathic pulmonary fibrosis (IPF) patients (24, 25). Boyang et al. (12) found that in connective tissue diseases, patients with the UIP pattern experienced accelerated FVC decline, reduced non-transplant survival, and diminished response to immunosuppression compared to those with NSIP or OP patterns. The presence of anti-Ro-52 antibodies (26) and elevated KL-6 levels (27–29) are also associated with more severe and progressive ILD. Our analysis uniquely links this high-risk antibody profile to a specific UIP phenotype, poorer pulmonary function, elevated pulmonary severity biomarkers, and a higher rate of cardiac involvement, thereby delineating a more meaningful high-risk patient phenotype. Notably, Cluster 2 (systemic inflammation subtype) was distinguished by a prominent association between anti-EJ antibodies and systemic features such as fever and Raynaud‘s phenomenon. This suggests that different anti-aminoacyl-tRNA synthetase (anti-ARS) antibodies may correlate with distinct systemic inflammatory phenotypes. The long-term implications of these phenotypes for ILD progression warrant further investigation. Cluster 1 is mainly characterized by anti-Jo-1 antibodies, with relatively mild pulmonary damage. According to literature reports, the prognosis may be relatively good. Patients in Cluster 3 have the shortest disease course but the most severe pulmonary involvement, suggesting that this group may present a more aggressive disease process, that is, severe pulmonary lesions occur at the early stage of the disease. This group is mainly positive for anti-PL-7/PL-12 antibodies. Previous studies have confirmed that such antibodies are associated with more rapidly progressive ILD. At the same time, this group has the highest proportion of cardiac involvement, further corroborating the severity and complexity of their conditions. Based on these characteristics, we speculate that Cluster 3 may have the worst treatment responsiveness, the highest recurrence rate and mortality rate. The characteristics of Cluster 2 are intermediate between the two. These inferences need to be further verified in future prospective longitudinal studies.

High-resolution computed tomography is essential for assessing interstitial lung disease, yet its interpretation can be subjective. The Warrick scoring system addresses this by providing a semi-quantitative method that grades both the type (e.g., ground-glass opacities, reticulation, honeycombing) and extent of lung abnormalities into a composite score, offering a more objective and reproducible measure of severity (30). Studies have demonstrated that the Warrick score correlates with pulmonary function tests and can be used to derive indices for alveolitis and fibrosis (31). Furthermore, recent evidence suggests that changes in the Warrick score over time may serve as a biomarker for predicting ILD progression (32, 33). In our study, a Warrick score ≥20 defined severe ASS-ILD. Patients meeting this threshold had a higher frequency of diffusion impairment, a greater proportion of UIP pattern, and elevated biomarkers of pulmonary severity, supporting the clinical validity of this cutoff in ASS-ILD. This quantitative tool allows for the comparison of pulmonary status both between different patients and at different time points in the same patient, serving as a key endpoint in clinical trials and longitudinal studies.

Our clustering results indicate that patients who predominantly exhibit the UIP phenotype within ASS-ILD are the oldest, and that age is an independent risk factor for severe ASS-ILD. Previous studies have identified age as an independent risk factor for IPF, where UIP serves as the core radiographic manifestation (34, 35). This strongly supports our findings and suggests that age may be associated with the development of the UIP phenotype—a pattern characterized by fibrosis as the primary radiographic feature. Mechanistically, aging is accompanied by telomere shortening, mitochondrial dysfunction, and cellular senescence in alveolar epithelial cells, which impair lung repair capacity and promote persistent fibroblast activation, leading to excessive extracellular matrix deposition (36). Consequently, this provides compelling evidence from a pulmonary fibrosis standpoint that age plays a contributory role in severe ASS-ILD. Furthermore, rash was found to be a protective factor against severe ASS-ILD. This aligns with reports in other autoimmune myositis syndromes, such as the subgroups of anti-MDA5 antibody-positive dermatomyositis described by Allenbach et al. (37). In that study, patients with joint involvement had the best prognosis, those with skin and muscle involvement had an intermediate prognosis, and those with rapidly progressive ILD (RP-ILD) had the poorest prognosis with high mortality. The “immune transfer hypothesis” provides a reasonable explanation: when the autoreactive immune response targets extrapulmonary tissues such as the skin, joints, and muscles simultaneously, the immune burden on the lungs may be partially reduced, resulting in a decrease in the degree of lung involvement. Our finding that rash is protective supports this concept, indicating that when ASS-related immune damage extends to tissues beyond the lungs, the associated pulmonary injury tends to be relatively milder. The complement system, which comprises over 30 proteins present in plasma and on cell membranes, plays a central role in innate immunity by recognizing and clearing pathogens, abnormal self-substances, and immune complexes (38). It also bridges and amplifies signals between innate and adaptive immunity (39). In autoimmune diseases, complement can be a double-edged sword: excessive activation contributes to tissue injury, whereas adequate complement levels are essential for efficient immune complex clearance. Low serum C3 may reflect ongoing consumption due to persistent immune complex formation and complement activation, which is often associated with more severe disease activity. Conversely, preserved C3 levels may indicate a greater functional reserve for clearing pathogenic autoantigens, thereby attenuating pulmonary inflammation and fibrosis (40). While the pathogenesis of ASS is not fully elucidated, it is broadly characterized as an autoimmune reaction against aminoacyl-tRNA synthetases in genetically predisposed individuals, often triggered by environmental factors (5). This reaction results in a systemic autoimmune disease that primarily affects the lungs, though it can involve multiple organs. Our findings indicate that complement C3 acts as a protective factor in severe ASS-ILD. The upper limit of the normal serum C3 level may reflect an enhanced ability of the complement system to clear the target autoantigen (i.e., aminoacyl—tRNA synthetase), thereby reducing subsequent abnormal immune activation and alleviating lung tissue damage. In summary, the three variables—rash, age, and complement C3—characterize ASS-ILD from three complementary aspects: extra-pulmonary disease manifestation, propensity for pulmonary fibrosis, and state of immune-inflammatory activation. The prediction model built on these variables therefore demonstrates strong predictive performance.

This study integrated multidimensional clinical, imaging, and serological data, revealing substantial heterogeneity within ASS-ILD. We identified three distinct clinical subtypes: joint-predominant, systemic inflammatory, and severe pulmonary. The severe pulmonary subtype—characterized by older age, anti-PL-7/PL-12 antibody positivity, a UIP pattern on HRCT, elevated KL-6 levels, and a high Warrick score—represents a high-risk subgroup that warrants particular clinical attention. Furthermore, older age, absence of skin rash, and serum complement C3 levels at the lower end of the normal range were identified as independent risk factors for severe ASS-ILD. These three variables were combined into a composite biomarker index that was developed and validated for risk stratification. Several limitations should be noted. First, the single-center, retrospective design may introduce selection bias. Second, although 100 patients were included, subdivision into three clusters led to limited sample sizes in some subgroups—especially the severe pulmonary cluster—which may affect the statistical power of subgroup comparisons. Third, although the clinical prediction model showed good discriminatory performance in our cohort, it requires validation in an independent, prospective external population to confirm its generalizability and clinical utility. Finally, this study has a cross—sectional design and fails to provide direct data on the long-term clinical trajectories of the three clustering groups. Our inferences about the prognosis of the groups, which are based on baseline characteristics and literature reports, need to be verified in future prospective cohort studies.

## 5 Conclusion

This study enhances the understanding of disease heterogeneity in ASS-ILD and underscores the importance of integrating multidimensional data for precise phenotypic classification. The identification of high-risk clinical subtypes and the development of a corresponding risk-prediction model offer a valuable framework for early risk stratification in clinical practice, support individualized therapeutic decision-making, and inform the design of future targeted studies. Further prospective, multicenter research is warranted to validate the stability of these subtypes, as well as to elucidate the distinct immunopathological mechanisms underlying each subtype.

## Data Availability

The original contributions presented in the study are included in the article/Supplementary material, further inquiries can be directed to the corresponding authors.
